# Supplementary material for: Completing the Genome Sequence of Chlamydia pecorum Strains MC/MarsBar and DBDeUG: New Insights into This Enigmatic Koala (Phascolarctos cinereus) Pathogen
Source: Pathogens. 2021 Nov 25;10(12):1543. doi: 10.3390/pathogens10121543 (PMC8703710; doi:10.3390/pathogens10121543)
Supplement: Supplementary file 1 [file pathogens-10-01543-s001.zip › pathogens-White2021_SupplementalData/Figure_S3.pdf]

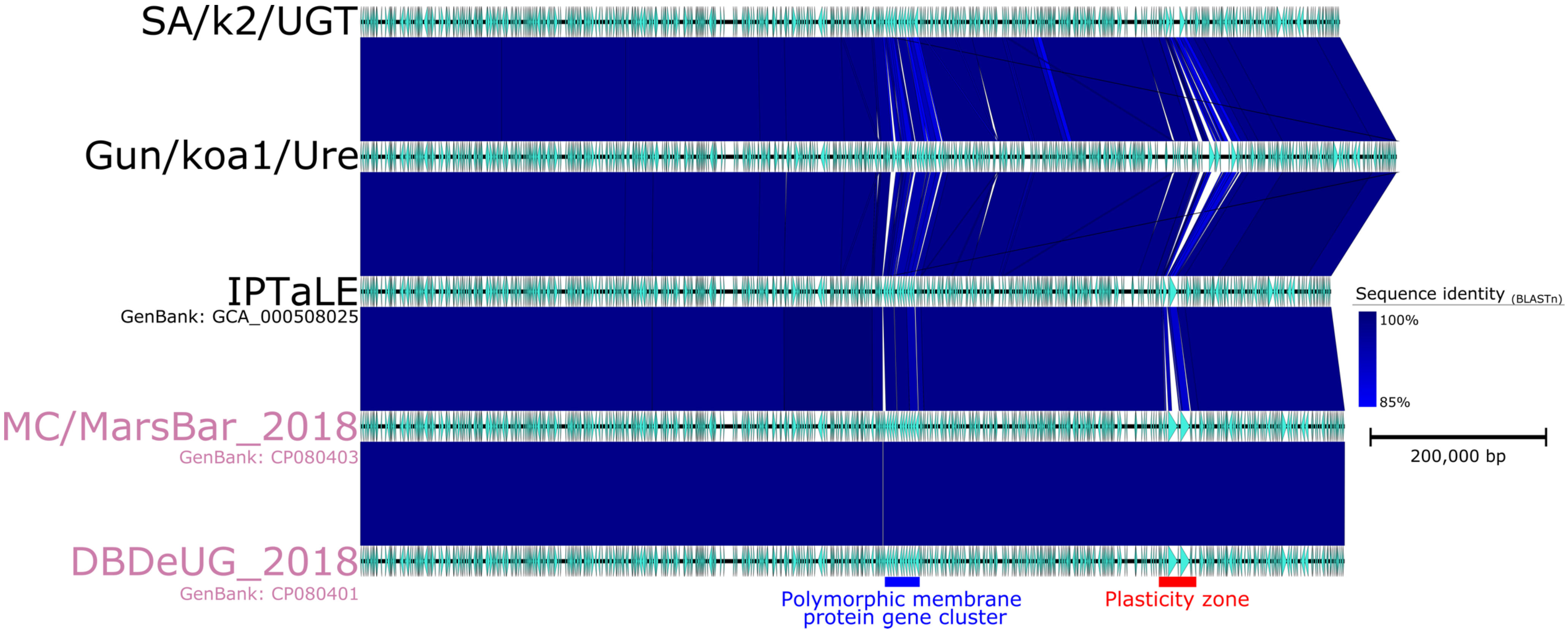


**Figure S3. Whole-genome nucleotide pair-wise comparisons of *Chlamydia pecorum* from koalas**. Blue shading indicates nucleotide identity between sequences according to BLASTn (85 to 100%). Plasticity zone (red) and major polymorphic membrane protein gene cluster (blue) are labelled. Strains Gun/koa1/Ure, IPTaLE, and SA/k2/UGT are represented as draft genomes. Image created using Easyfig v2.2.2.
