# Supplementary material for: Completing the Genome Sequence of Chlamydia pecorum Strains MC/MarsBar and DBDeUG: New Insights into This Enigmatic Koala (Phascolarctos cinereus) Pathogen
Source: Pathogens. 2021 Nov 25;10(12):1543. doi: 10.3390/pathogens10121543 (PMC8703710; doi:10.3390/pathogens10121543)
Supplement: Supplementary file 1 [file pathogens-10-01543-s001.zip › pathogens-White2021_SupplementalData/Figure_S2.pdf]

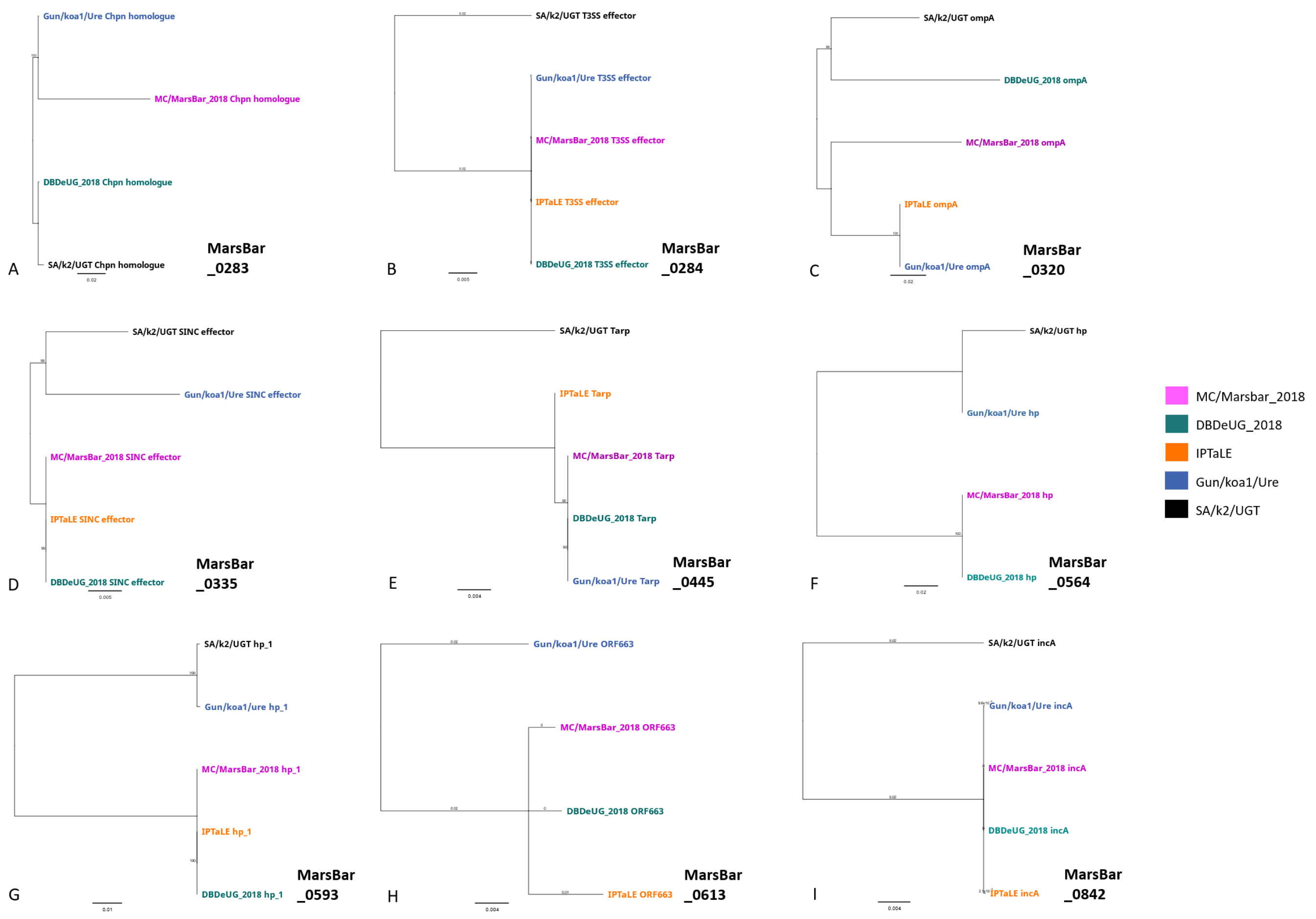

Figure S2. Genetic diversity of koala *Chlamydia pecorum* selected polymorphic loci. Mid-point rooted Neighbor-Joining trees, constructed using HKY+G nucleotide substitution model using Geneious Tree Builder as implemented in Geneious Prime, from the koala strains polymorphic genes alignments. Trees are as follows from alignments of A: Chpn 76kDA homologue (locus tag: MarsBar_0283); B: T3SS effector (locus tag: MarsBar_0284); C: *ompA* (locus tag: MarsBar_0320); D: T3SS effector SINC (locus tag: MarsBar_0335); E: T3SS effector Tarp (locus tag: MarsBar_0445); F: Conserved hypothetical protein (hp) (locus tag: MarsBar_0564); G: Conserved hypothetical protein (hp1) (locus tag: MarsBar_0593); H: ORF663 homologue (locus tag: MarsBar_0613); and I: inclusion membrane protein A (*incA*) (locus tag: MarsBar_0842). Support values (using 1,000 Bootstraps) are displayed on the nodes, and scale represent the rate of nucleotide substitution per site. Each koala strain is denoted by colours outlined in the legend.
