# Supplementary material for: Completing the Genome Sequence of Chlamydia pecorum Strains MC/MarsBar and DBDeUG: New Insights into This Enigmatic Koala (Phascolarctos cinereus) Pathogen
Source: Pathogens. 2021 Nov 25;10(12):1543. doi: 10.3390/pathogens10121543 (PMC8703710; doi:10.3390/pathogens10121543)
Supplement: Supplementary file 1 [file pathogens-10-01543-s001.zip › pathogens-White2021_SupplementalData/Figure_S1.pdf]

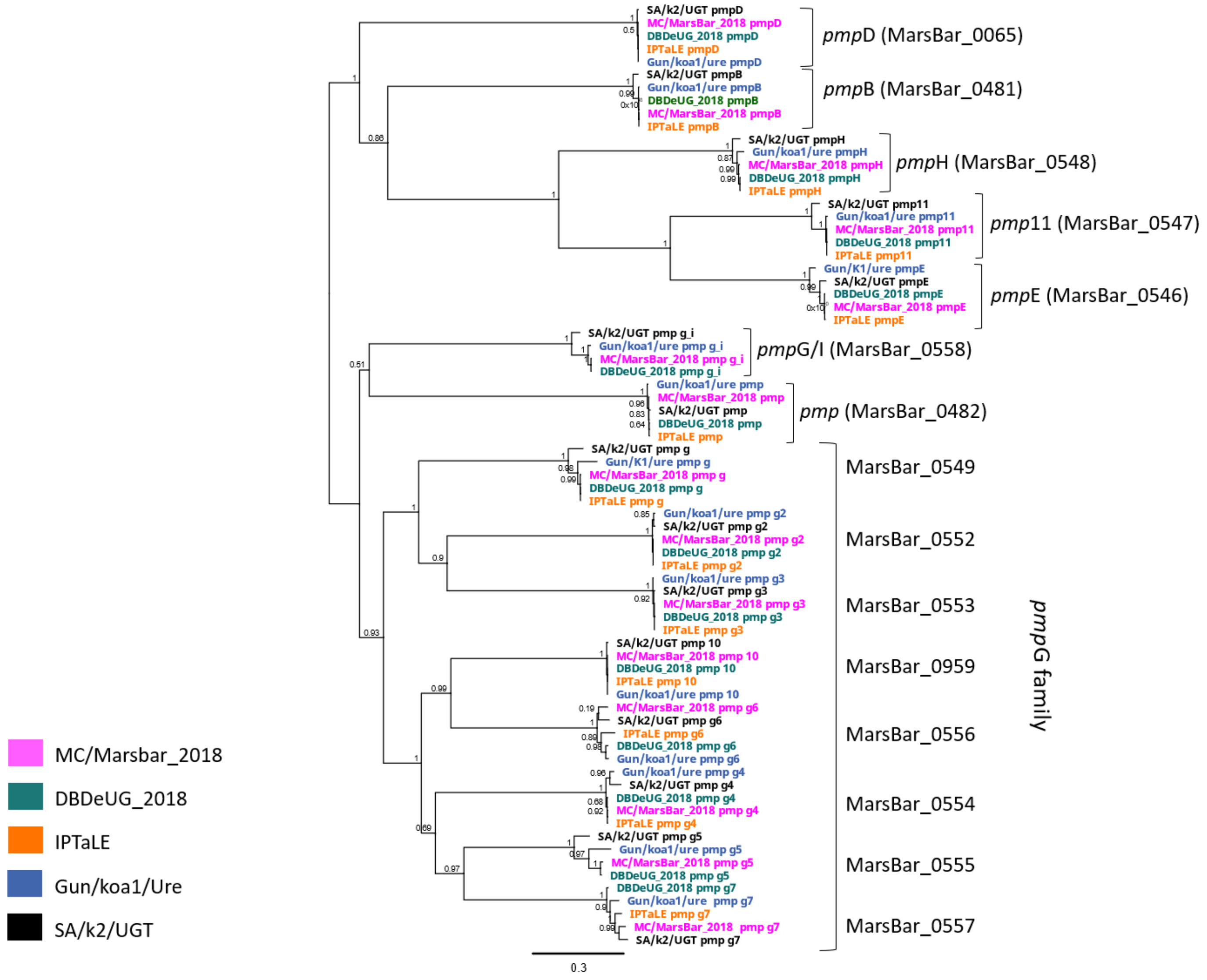

Figure S1. Genetic diversity of koala *Chlamydia pecorum* polymorphic membrane proteins (*pmp*). Mid-point rooted approximate maximum likelihood tree, constructed using GTR+G nucleotide substitution model using FastTree 2.1.11 as implemented in Geneious Prime, from the koala strains *pmp* genes alignment. Support values (using 1,000 resamples) are displayed on the nodes, and scale represent the rate of nucleotide substitution per site. Each koala strain is denoted by colours outlined in the legend, and locus tag in Mc/Marbar_2018 is displayed next to the corresponding clade.
